# Double-edged sword of JAK/STAT signaling pathway in viral infections: novel insights into virotherapy

**DOI:** 10.1186/s12964-023-01240-y

**Published:** 2023-10-02

**Authors:** Mohamad Mahjoor, Golnaz Mahmoudvand, Simin Farokhi, Alireza Shadab, Mojtaba Kashfi, Hamed Afkhami

**Affiliations:** 1 Cellular and Molecular Research Center, Qom University of Medical Sciences, Qom, Iran; 2 Department of Immunology, Faculty of Medicine, Iran University of Medical Sciences, Tehran, Iran; 3 Student Research Committee, USERN Office, Lorestan University of Medical Sciences, Khorramabad, Iran; 4 Department of Immunology, School of Medicine, Semnan University of Medical Sciences, Semnan, Iran; 5 Iran University of Medical Sciences, Deputy of Health, Tehran, Iran; 6 Nervous System Stem Cells Research Center, Semnan University of Medical Sciences, Semnan, Iran; 7 Department of Medical Microbiology, Faculty of Medicine, Shahid Beheshti University of Medical Sciences, Tehran, Iran; 8 Department of Medical Microbiology, Faculty of Medicine, Shahed University, Tehran, Iran

**Keywords:** JAK/STAT signaling pathway, Antiviral, Inflammation, Interferon, Cytokine

## Abstract

The Janus kinase/signal transducer and activator of transcription (JAK/STAT) is an intricate signaling cascade composed of various cytokines, interferons (IFN, growth factors, and other molecules. This pathway provides a delicate mechanism through which extracellular factors adjust gene expression, thereby acting as a substantial basis for environmental signals to influence cell growth and differentiation. The interactions between the JAK/STAT cascade and antiviral IFNs are critical to the host’s immune response against viral microorganisms. Recently, with the emergence of therapeutic classes that target JAKs, the significance of this  cascade has been recognized in an unprecedented way. Despite the functions of the JAK/STAT pathway in adjusting immune responses against viral pathogens, a vast body of evidence proposes the role of this cascade in the replication and pathogenesis of viral pathogens. In this article, we review the structure of the JAK/STAT signaling cascade and its role in immuno-inflammatory responses. We also highlight the paradoxical effects of this pathway in the pathogenesis of viral infections.

Video Abstract

**Graphical Abstract:**

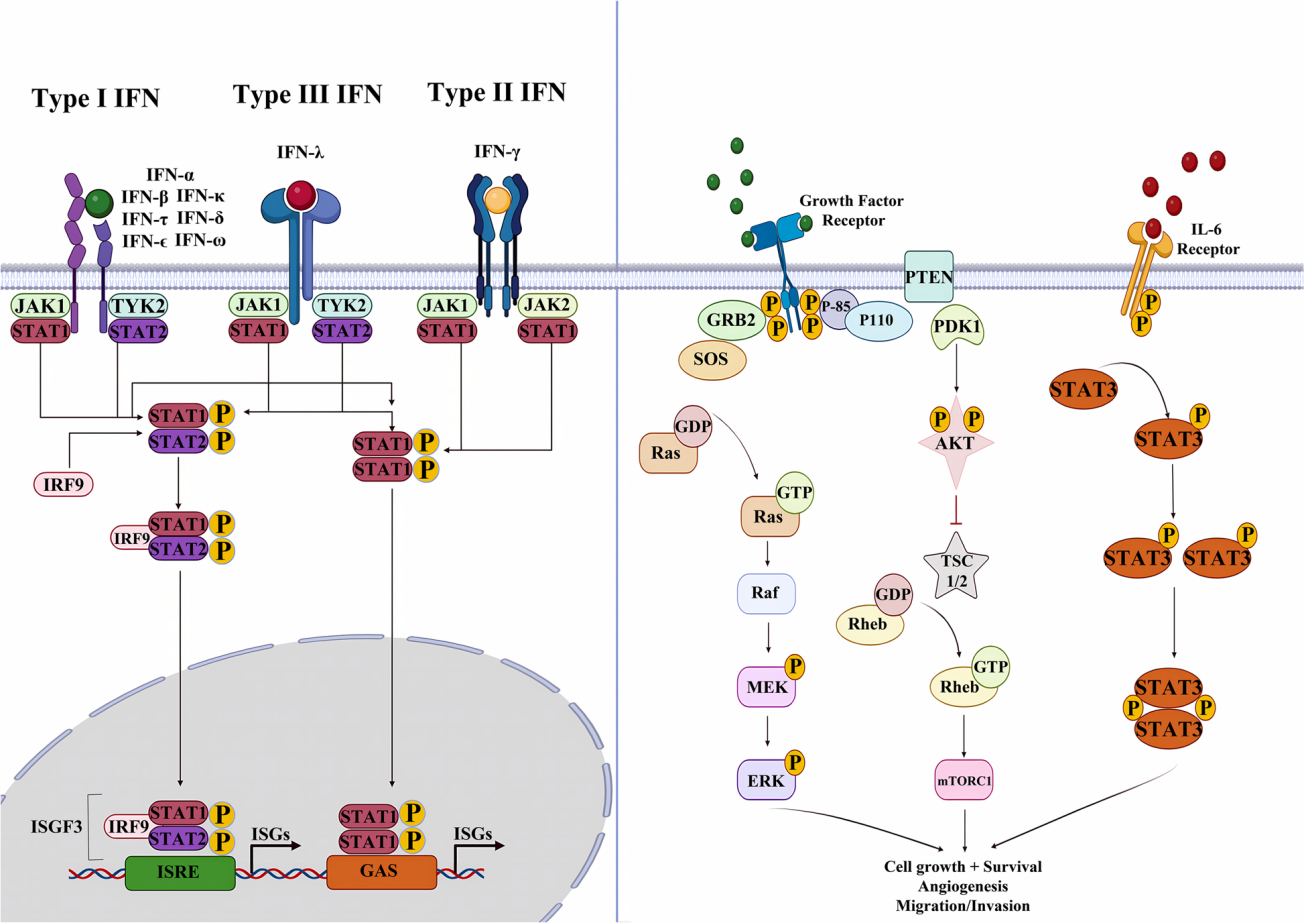

**Supplementary Information:**

The online version contains supplementary material available at 10.1186/s12964-023-01240-y.

## Introduction

The structure of antiviral immune signaling is nuanced, multi-waved, and interlinked. Innate immunity serves as the frontline component of the immune system in recognizing and eliminating viral infections [1, 2]. Following the entrance of viruses into target cells, pattern-recognition receptors (PRR) identify the viral components and induce interferon (IFN) synthesis [3]. These cytokines employ various mechanisms to communicate and exert their effects. The Janus kinase-signal transducer and activator of transcription (JAK/STAT) cascade constitutes an important intracellular mechanism cytokines use for signaling [4, 5]. Historically, investigations on gene provocation by IFNs facilitated the recognition of the JAK/STAT pathway [6]. Since then, several cross-talks between this pathway and the immune system have been discovered [7]. This cascade acts as a transit center for cytokine production and is utilized by numerous pro-inflammatory molecules to facilitate their downstream effects and invoke gene transcription [8]. The JAK/STAT pathway is triggered once the released IFNs engage with their specific receptors [3], causing the release of pro-inflammatory cytokines and the generation of downstream antiviral IFN-stimulated genes (ISGs). Following this process, an antiviral environment forms that stops virus reproduction and triggers the adaptive immune response, and attracts other immune cells to the infection site. Consequently, this process leads to the rapid elimination of viruses from infected cells [9, 10]. Besides IFNs, at least 50 cytokines and growth factors, including hormones, interleukins (ILs), and colony-stimulating factors, have been found in the JAK/STAT signaling apparatus [11].

As the stimulation of the antiviral response by IFN seriously endangers virus survival, viruses have adopted specific strategies such as proteasomal degradation and dephosphorylation to target the JAK/STAT pathway, thereby fighting against the host’s innate immune system. The vast bulk of viruses that exploit the JAK/STAT pathway in this manner affect STAT1 and STAT2. On the other hand, most viruses appear to prefer the transcriptional blocking of target gene expression, which prevents nuclear translocation and the production of the transcription complex ISGF3. Some viruses may also activate the suppressor of cytokine signaling (SOCS) genes, which prevents the tyrosine phosphorylation of STATs and controls the pathways [11, 12].

Despite the vital role of the JAK/STAT pathway in boosting immune responses against viral pathogens, recent findings allude to the positive effects of this pathway on the replication and pathogenesis of viral infections [13]. Components of the JAK/STAT cascade exert pro- or antiviral impact depending on the type of virus and host cell and collectively play a determining role in the cross-talk between viral pathogens and their hosts. Researchers have used this concept to develop novel antiviral medications by modifying the genes and molecules involved in the cascade.

This article outlines the dual role of the JAK/STAT pathway in viral infections.

## JAK /STAT pathway

The JAK/STAT cascade represents one of the numerous intracellular mechanisms cytokines use to signal. Particularly, cytokines linking to type I/II receptors exploit the JAK/STAT pathway to exert their effects [4]. The JAK family consists of four cytoplasmic tyrosine kinases (JAK1, JAK2, JAK3, and TYK2) that connect to the intracellular regions of many transmembrane cytokine receptors. The STAT family, which consists of the seven intracellular transcription factors STAT1, STAT2, STAT3, STAT4, STAT5a, STAT5b, and STAT6, is connected to their function. These proteins are implicated in cell-mediated immunity, proliferation, differentiation, and apoptosis and are composed of an N-terminal domain, a DNA-binding domain, and a C-terminal transactivation domain [47]. Different homology domains, including 4.1, ezrin, radixin, moesin (FERM), Src homology 2 (SH2), kinase, and pseudokinase form JAKs. The kinase and pseudokinase domains are constructed by JH1 and JH2, respectively [48, 49]. The N-terminus part of the members of the JAK family includes FERM and SH2 domains that help JAK to interact with cytokine receptors through their cytoplasmic tails [50]. The attachment of the ligand to the cytokine receptor alters the orientation of the receptor/JAK dimers, putting the JAK near its partner in the dimer at JH1 to initiate transphosphorylation. Compared to JH1, the JH2 domain shows 10% catalytic activity [51]. As the loss of JH2 results in continuous action, it is supposed to play an auto-inhibitory role [52]. In response, stimulated JAKs phosphorylate residues on the cytoplasmic domain of the cytokine receptor to provide "docking sites" for recruiting downstream proteins containing SH2 domains, such as the STAT protein group [48]. Since the receptors have distinct affinities for the JAK group protein they employ as a signaling effector, a strong link forms between the receptor and the particular JAK proteins triggered [8, 53].

Members of the STAT protein class are then recruited to continue this signaling cascade. Although deactivated STATs may be found in the cytoplasm, the noncanonical activation mechanism suggests that nonphosphorylated STATs also exist in the nucleus [54]. STATs, as their title implies, function as both signal transducers and transcription factors. However, unlike other transcription factors, STATs have two structural features that set them apart: an SH2 domain and a perfectly conserved C-terminal tyrosine residue [55]. This tyrosine residue is the target for phosphorylation by stimulated JAKs. Phosphorylation triggers STATs to link with one another through their SH2 domains and create stable homodimers or heterodimers [56]. Similar to JAKs, members of the STAT family react specifically to a limited spectrum of stimuli and receptors. STAT3 is the only STAT known to be triggered by extracellular SRC and EGFR [57, 58]. Negative regulators, such as SH2-containing protein tyrosine phosphatase (SHP) and SOCS proteins, may also be capable of switching off the JAK/STAT activation [59]. CIS, SOCS1, SOCS2, SOCS3, SOCS4, SOCS5, SOCS6, and SOCS7 are all members of the SOCS family, which are intracellular proteins. To downregulate the JAK/STAT signaling pathway, activated STATs dimerize and infiltrate the nucleus, inducing the production of SOCS, which then binds to phosphorylated JAK and its receptor. The JAK/STAT pathway is inversely regulated by SOCS via three mechanisms. As CIS binds persistently to the tyrosine-phosphorylated β chain of the IL-3 receptor and the tyrosine-phosphorylated EPO receptor, it blocks STAT recruitment to the receptor [60]. By binding selectively to either JAK or its receptor, SOCS blocks the kinase function of JAK. By way of illustration, SOCS3 binds to both JAK and its receptor gp130, which is part of the IL-6 family cytokine-receptor complex. Once the SH2 domain of SOCS3 is attached to phosphorylated Tyr759 of gp130, Ig-like receptors (KIR) of SOCS3 engage with gp130-related JAK in a nonphosphorylation-dependent way. When SOCS3 binds to JAK, it covers the protein's substrate-binding groove, blocking JAK/STAT complex [61]. The SH2 domain of SOCS1 might interfere with the activation loop of JAKs, and SOCS1 has the ability to block JAK tyrosine kinase function via KIR [62]. The elongation protein B/C complex communicates with the SOCS proteins via the C-terminal SOCS box, and cullin5 joins the SOCS3 E3 ubiquitin-linked enzyme complex simultaneously [63].

JAK/STAT signaling cascade commences when a ligand, including growth factors, interferons, or interleukins, binds to particular transmembrane receptors and activates JAK. Many receptors have been linked to JAK/STAT cascade activation, with cytokine receptors being the major transmembrane receptor family related to JAK activation [64, 65]. Cytokine receptors induce the JAK/STAT pathway via various combinations of JAK and STAT components, revealing the versatility of this system. Interleukin (IL) receptors, interferon (IFN) receptors, and colony-stimulating factor receptors are the receptors in this family that are associated with JAK activation (CSFRs). Among IL receptors, gp130 subunit and receptors for IL-2, IL-3, IL-4, IL-6, IL-7, IL-9, IL-10, IL-11, IL-12, IL-13, IL-15, IL-20, IL-21, IL-22, IL-27, IL-31, and Leptin have been noted to activate selected components of the JAK family. Nevertheless, while JAK1 appears to be a popular factor, a variety of compositions in downstream effectors have been recognized. By way of illustration, heterodimerization of the IL-2Rb and gc cytoplasmic domains activates JAK1 and JAK3, with JAK1 interacting with IL-2Rb and JAK3 with gc [66]. Communication between IL-2 and its receptor mostly activates STAT5, but STAT3 and STAT1 are affected to a lesser extent [67]. EPOR is a hormone receptor with extra-cytoplasmic structural properties similar to the cytokine receptor family [68]. In 1994, D’Andrea and Barber showed that EPOR activation could cause an instant, dose-dependent JAK2 phosphorylation [69]. Meanwhile, simulating the IL-3 receptor by the proteasome inhibitor N-acetyl-L-leucinyl-L-leucinyl-norleucinal (LLnL) has resulted in extended activation of JAK 1 and 2, as well as steady phosphorylation of STAT5 [70]. It is postulated that cytokine receptors selectively employ one or a particular mixture of JAK family proteins [71]. Notwithstanding, the exact mechanism of this selectivity remains to be discovered. Notably, IL-4R [70] and IL-13R [72] are the only cytokine receptors capable of signal transduction to STAT6. STAT6 has distinct activities in various cell types and induces the transcription of a distinct collection of proteins in T cells relative to non-lymphocyte cells [73]. The IL-5 receptor is fundamental to the functioning of eosinophils, which are multifunctional granulocytes related to asthma and inflammation [74]. STAT1 and STAT5 are triggered by the signaling initiated by this receptor, but IL-6 and IL-10 primarily activate STAT3, which might cause varying effects [75, 76]. STAT1 and STAT3 are triggered by an IL-6R signal; nonetheless, distinct cell types exhibit a significant preference for one STAT over the other. SOCS3 is a protein that can be activated by STAT signaling from several cytokine receptors, and it inhibits the production of IL-6R through feedback. Much enhanced STAT3 activation can be observed in the lack of SOCS3 [77]. SOCS3 suppresses Th1 cells and increases Th2 synthesis by preventing the activation of STAT4 by IL-12. The absence of SOCS3 may also suppress the formation of Th1 cells and Treg cells by boosting the synthesis of IL-10 and transforming growth factor (TGFβ) [78]. Nevertheless, STAT1 stimulation is not similarly inhibited; hence, in the presence of SOCS3, the pathway triggered by IL-6R flips from STAT3 to STAT1 to a certain degree [79]. Although IL-10R signaling mimics the IL-6R pathway, IL-10 STAT3 activation promotes the transcription of a distinct array of proteins directly associated with suppressing inflammatory responses [80]. As part of the collaboration between SMAD3 and STAT3, STAT3 may also inhibit SMAD3–SMAD4 complex formation and reduce SMAD3-DNA binding. SMAD3 may also bind PIAS3 to STAT3, thereby limiting STAT3 activity [81]. SMAD3 and STAT3 phosphorylation states dictate whether their connection is collaborative or oppositional [82]. TGFβ inhibits IL-12-mediated JAK2 and TYK2 tyrosine phosphorylation, as well as STAT3 and STAT4 activation in T lymphocytes, thereby reducing T-cell proliferation and IFN-γ production [83]. IL-12R and IL-23R are similar in structure, utilize a similar signaling pathway, and belong to the group of cytokine receptors whose signal transduction requires TYK2. IL-31 is primarily generated by CD4 + T cells and pertains to the gp130/IL-6 cytokine family. IL-31R stimulates the JAK/STAT, PI3K/AKT, and MAPK signaling cascades and affects various cell types [84]. What distinguishes between the pathways triggered by type I (IFN-α and β) or type II (IFN-γ) IFN receptors is TYK2. IFNaR1 and R2 (b) are connected with TYK2 and JAK2, whereas IFNgR1 and R2 stimulate JAK1 and JAK2, respectively [85]. Briscoe et al. It has been shown that JAK1-negative U4A cells exhibit a limited response to IFN-γ, but JAK2-negative g2A cells failed to react at all to IFN-γ [86]. An activated IFN receptor can induce different intracellular proteins from other signaling cascades, such as MAP kinase, PI3-K, CaMKII, and nuclear factor‐κB (NF‐κB) [87].

Granulocyte Colony Stimulating Factor (G-CSF) and Granulocyte/Macrophage Colony Stimulating Factor (GM-CSF) were shown to interact with JAK/STAT cascade activation. G-CSFR predominantly activates JAK2 and STAT3 and can be found in both normal and malignant tissue [88]. Myeloid progenitors, mature monocytes, neutrophils, eosinophils, basophils, and dendritic cells express GM-CSF and help the immune system fight against bacterial diseases [87]. GMCSFR can trigger the activation of JAK2, while STAT5 is the major component of the STAT family to be affected by this pathway [89]. Fibroblast growth factor receptor (FGFR), vascular endothelial growth factor receptor (VEGFR), and platelet-derived growth factor receptor (PDGFR) also showed cross-talks with this pathway. FGFR has the capacity to stimulate STAT1 and STAT3 via JAK2 [90]. It is believed that tyrosine phosphorylation of STAT3 through this receptor occurs in a JAK-dependent way, which is based on the production of a complex by JAK2 and Src with FGFR1 [89]. Hormone receptors are also linked to JAK/STAT pathway. Besides EPOR, the prolactin receptor (PRLR) can aid in activating this pathway. In 1997, Pezet and colleagues demonstrated that prolactin binding to its receptor leads to the dimerization of JAK2, which has a fundamental association with this receptor [91]. As a matter of fact, JAK/STAT is the primary signaling pathway induced by PRLR [92]. The growth hormone receptor is another hormone receptor involved in JAK2 activation [93]. Although JAKs have well-established roles in stimulating STATs in response to cytokine activation, a novel role for JAK2 in the nucleus was later revealed, in which JAK2 contributes to epigenetic control of gene transcription by phosphorylation of tyrosine 41 on the histone protein H3. This noncanonical pathway is preserved with JAK1, which is triggered by the autocrine cytokines IL6 and IL10 in activated B-cell-like diffuse large B-cell lymphoma, a difficult-to-treat malignancy with a dismal prognosis [94].

### JAK/ STAT pathway, inflammation and the immune system

#### JAK/ STAT pathway and immune system

**Fig. 1:**
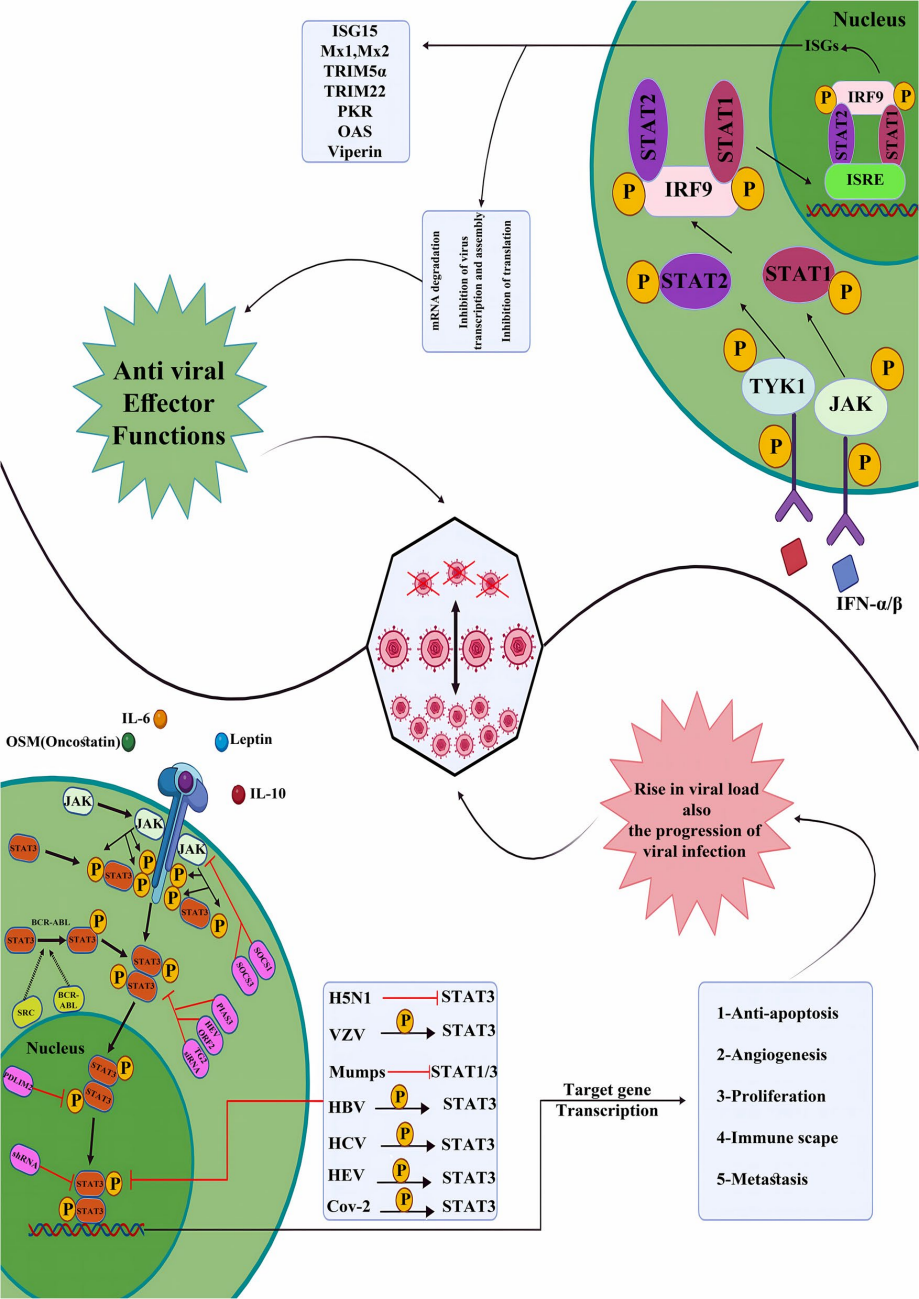
Cytokines secreted in battlefields provoke both innate and adaptive responses against pathogens

Cytokines released in the infected area can induce and regulate both innate and adaptive defenses against foreign invaders [95]. These activities mainly depend on the certain receptors present on the surface of target cells. Following the attachment of cytokines to their related receptors, cascades of intracellular signaling provoke defense mechanisms against invaders [96]. One of the commonly activated intracellular signalings is the JAK/STAT pathway [97]. Every cytokine is inclined to induce a particular STAT; nevertheless, the interaction between different cytokines and all types of STAT exists to varying extents [98]. Some genes downstream of STATs are principal determiners of the signaling cross-talks [99]. The mechanisms by which STATs interact with parts of the immune system, particularly cytokines, have been examined [98]. Once a cell is exposed to a particular cytokine, subsequent provocation with the same or another type of cytokine might lead to both antagonistic or synergistic outcomes, providing the cellular basis and the type of cytokines entangled [100]. As an illustration, pretreatment with IFN-γ significantly increases the sensitivity of cells to IFN-α, primarily due to the overexpression of STAT1 and IRF9 incited by IFN-γ. Similarly, pre-exposure to slight amounts of IFN-γ can promote the upcoming IFN-γ functioning during macrophage activation [99] (Fig. 1). Additionally, ISG15, which is encoded by a type I IFN, regulates members of the JAK-STAT cascade via protein ISGylation, causing an augmented IFN-α response [101]. Dysregulation of the JAK-STAT cascade occasions several types of immune disorders. Mutations of the Jak-3 gene were attributed to autosomal recessive severe combined immune deficiency (SCID) when decreased levels of Jak-3 were detected in the affected patients. SCID results from mutations in the common γ-chain (a component of IL-2, IL-4, IL-7, IL-9, and IL-15 receptors). This disorder is highlighted by the lack of circulating T cells while B lymphocytes are present [102]. JAK/STAT signaling is also responsible for the immune regulatory responses entangled in tumor cell distinction and tumor-induced immune escape. Onco-suppressive immune processes are mediated mainly by STAT1 and STAT2 promotion of type I and II IFNs, while STAT3 activity leads to immunosuppression and increased survival of tumor cells [11]. Given the crucial role of the JAK/STAT cascade in the pathogenesis of several immune disorders, numerous novel therapeutic options have focused on inhibiting the JAK/STAT pathway. In a patient with glioblastoma, suppression of the JAK/STAT cascade salvaged T-cell functionality *in-silico* multidimensional model and in vivo, indicating the potential of JAK-inhibitors to enhance T-cell activation in myeloid and glial cells [103]. Some other medications blocking JAK/STAT-activating cytokines include the anti-IL-6 antibody (siltuximab), used for the management of Castleman’s disease, and anti-IL-6 receptor antibody (tocilizumab), used for the treatment of RA and juvenile idiopathic arthritis [104] (Tables 1 and 2).

**Table 1:** Inducers of STAT family components

Cytokine	STAT component	Transcription factor	Gene
IFNα, IFNβ, and IFNγ	STAT1	ISGF3, GAF	ISRE, GAS [14]
IFNα and IFNβ	STAT2	ISGF3	ISRE [15]
IL-6, IL-10, IL-11, IL-21, IL-23, LIF, and OSM	STAT3	NF-κB	ISRE [16, 17]
IL-12, IFN-α	STAT4	IRF-1	GAS [18]
IL-2, IL-3, IL-5, IL-7, IL-15, GM-CSF	STAT5A and STAT5B	Eos, GATA3	TH2 gene, Il4, GAS [19–21]
IL-4 and IL-13	STAT6	GATA3	GATA3, Il4 [21]

*IFN* Interferon, *ISGF3* Interferon Stimulated Gene Factor 3, *GAF* GAGA factor, *ISRE* Interferon-sensitive response element, *GAS* Gamma interferon activation site, *IL* Interleukin, *LIF* Leukemia inhibitory factor, *OSM* Oncostatin M, *NF-κB* Nuclear factor kappa-light-chain-enhancer of activated B cells, *IRF-1* Interferon regulatory factor 1, *GATA3* GATA Binding Protein 3, *GM-CSF* Granulocyte–macrophage colony-stimulating factor

**Table 2:** Overview of inhibitors of the JAK/STAT signaling pathway and their application in the treatment of immune/inflammatory diseases

Inhibitor	Component affected	Indication	Effects
Ruxolitinib	JAK1/JAK2	Psoriasis Graft versus host disease	Improves skin lesions by inhibiting the infiltration of Th1 and Th17 cells [22]
Baricitinib	JAK1/JAK2	RA Psoriasis Alopecia areata	Improves disease activity in RA refractory to conventional medication Ameliorates skin involvement in immune diseases [23]
Tofacitinib	JAK1, JAK2, JAK3	RA Spondyloarthropathy Juvenile idiopathic arthritis Psoriasis Transplant rejection Ulcerative colitis	Prevents the progression of structural joint disease Blocks STAT phosphorylation in keratinocytes affected by psoriasis Conserves the remission of ulcerative colitis [23]
Decernotinib	JAK3	RA	Improves signs and symptoms of RA [24]
Peficitinib	JAK3/JAK1	RA Psoriasis	Ameliorates symptoms in moderate to severe RA [25] Has shown some efficacy in plaque psoriasis [26]
Filgotinib	JAK1	RA Crohn’s disease	Ameliorates RA signs and symptoms and inhibits its progression [27] Leads to clinical remission of Crohn’s disease [28]
Itacitinib	JAK1	Psoriasis RA	Significant improvements in chronic plaque psoriasis [29] Rapidly ameliorates symptoms of RA [30]
Upadacitinib	JAK1	RA	Improves disease activity [31]

*RA* Rheumatoid arthritis

### JAK/ STAT pathway and inflammation

Macrophages differentiate into diverse phenotypes, including pro-inflammatory M1 and anti-inflammatory M2, owing to the encompassing microenvironment. M1/M2 balance is essential for maintaining the functionality of the immune system. In vivo and in vitro studies have proposed the role of the JAK/STAT pathway in Macrophage polarization. Tyrosine phosphorylation of STAT-6 and JAK-1 mediates M2 activation [105]. Constant activation of JAK/STAT signaling is seen in abnormal conditions and usually leads to long-term inflammation and inflammation-mediated tumor development in several organs. It is also assumed that medications that suppress JAK/STAT signaling might be beneficial for inhibiting autoimmune-driven inflammatory processes along with the progression of chronic inflammation [106]. For example, the JAK inhibitor JTE-052 has shown a negative influence on antigen-specific T-cell stimulation and inflammation in contact hypersensitivity and irritant contact dermatitis, among other dermatological inflammatory disorders [107]. Furthermore, JAK inhibitors VX-509 and R-348 have been observed to suppress inflammatory responses and improve symptoms of psoriasis [108]. The activities of the JAK/STAT cascade have also been detected in neutrophils. Under activation of neutrophils by GM-CSF, JAK2 and the downstream STAT3/STAT5 pathway are activated, leading to NLRP3 protein expression and IL-1β release. Inhibition of this pathway is effective in the control of rheumatoid synovitis [109]. NLRP3 might be induced by both exogenous and endogenous triggers, such as urate and cholesterol crystals, resulting in severe inflammatory disorders like atherosclerosis, cardiovascular disease, and gout [110]. The JAK/STAT pathway is considered a pivotal cascade in developing inflammatory bowel disease (IBD). A study on intestinal inflammation showed that luteolin, a natural flavonoid, has demonstrated promising results by inhibiting the JAK/STAT pathway [111]. Moreover, G-CSF induces neutrophil differentiation and activation through JAK1/2 and STAT3 [112]. The role of this pathway has also been suggested in the pathogenesis of inflammatory joint diseases such as osteoarthritis. JAK2/STAT1/2 signaling has appeared to participate in Matrix metalloproteinase-13 induction in IL-1β provoked chondrocytes [113]. Studies on mice with retinitis pigmentosa have shown that JAK2/STAT3 pathway participates in microglial activation of inflammatory factors, including TNF-α, IL-6, MCP-1, ICAM, Arg1, IL-4, and IL-13 [114]. In cardiac tissue, JAK/STAT pathway mediates M1 macrophage polarization and myocardial ischemia/reperfusion injury [115]. In low-grade chronic inflammation commonly observed in older adults, a pan-cell type deficiency occurs in the JAK/STAT cascade, predisposing them to acute inflammation [116].

### JAK/ STAT pathway and immune system and inflammation

Inflammatory signaling pathways, namely NF‐κB, JAK/STAT, and mitogen‐activated protein kinases (MAPKs) constitute the chief pathways in enhancing and adjusting inflammatory responses in the immune system [117]. Improper activation or obliteration of the JAK/STAT cascade is a sign of the inflammatory response. This pathway is a part of innate and adaptive immune defense in inflammatory diseases [118]. In this regard, the role of this cascade in the adjustment of innate immunity in neuroinflammatory conditions is an excellent example. Pro-inflammatory macrophages are present in CNS lesions secondary to multiple sclerosis (MS) [119]. High concentrations of STAT1 and STAT6, as well as pro-inflammatory macrophages, have been detected in MS cases with protein tyrosine phosphatase SHP-1 defect [120]. In experimental autoimmune encephalomyelitis, IFN-γ is released by autoreactive Th1 cells through STAT4, consequently activating pro-inflammatory macrophages via STAT1. Also, Th17 cells synthesize GM-CSF, which leads to the pro-inflammatory polarization of macrophages via JAK2/STAT5 [121]. Innate immunity is also involved in the pathogenesis of IBD [122]. In a mouse model, the increased expression of IFNγ, IL22, and IL17, along with activation of JAK/STAT signaling were detected, confirming cytokine-mediated inflammation in colitis [123]. Rheumatoid arthritis (RA) is a widespread disease caused by aberrant immune-inflammation interactions. A myriad of immune cells are responsible for the pathogenesis of RA. Several immune cells from both innate and adaptive systems are involved in the occurrence of inflammation in the synovium [124]. JAK1, JAK2, JAK3, and TYK2 have a vital role in the cytokine pathway involved in the pathogenesis of RA. Activated JAKs induce STAT1, 2, 3, 4, 5A, 5B, and 6 to transfer to the nucleus and influence their target genes, with STAT 3 having the leading effect on organs involved by RA [125]. In spinal cord injury, IL-6R/JAK/STAT signaling mediates the immune-inflammatory process via the expression of iNOS and TNF-α, among others, negatively affecting the recovery of the spinal cord nerve [126]. An intact JAK/STAT signaling cascade is crucial for immune cells. Any deficiency in this system might lead to different immune disorders. In particular, the formation and well-functioning of T cells depend on JAK/STAT signaling, as mutations of the Jak-3 result in a lack of T cells [127].

### JAK/STAT pathway and its role in viral diseases

IFN-α is a potent antiviral cytokine that serves an undeniable role in the host immune response against viral pathogens. As mentioned earlier, soon after being expressed, IFN-α engages with the JAK/STAT pathway. This cross-talk drives the expression of several effector genes, namely ISGs, which are fundamental to a proper effector mechanism and eventually the omission of viruses [128]. A similar mechanism exists for IFNγ; however, while IFNs α and β are substantially responsible for viral defense, IFNγ can exert antiviral effects [129]. While some ISGs function as PRRs, increasing viral recognition and immune cell recruitment, others affect the viral life cycle, preventing subsequent viral infection [130]. Myxovirus resistance (Mx) genes are considered the most widely researched ISGs implicated in viral inhibition. There are two major types of Mx proteins, commonly referred to as Mx1 and Mx2. Research indicates that Mx1 is essential in capturing viral nucleocapsids as they enter the cell, preventing them from targeting their intended cellular destination. Mx2 acts similarly to Mx1 by identifying and isolating viral components, blocking the virus's life cycle [131]. The IFN-induced transmembrane (IFITM) protein is another prominent antiviral ISG product. This family consists of four members: IFITM1, IFITM2, IFITM3, and IFITM5. According to research, IFITM proteins can limit the reproduction of a variety of pathogenic viruses, particularly enveloped viruses [132]. Since IFITM proteins are primarily found in late endosomes and lysosomes, they are particularly active against viruses that employ these routes to enter the host cell environment [133].

2′-5′-oligo-adenylate synthetase (OAS) and protein kinase R (PKR) are two families belonging to ISGs that are involved antiviral process through the suppression of protein synthesis. OAS proteins possess a solid capacity to generate distinct 2′-5′-oligomers from ATP by synthesizing 2′,5′-linked phosphodiester linkages. These oligomers activate a dormant form of RNase L, resulting in viral and host RNA breakage [134]. Akin to OAS, the PKR family obstructs protein production in dsRNA-treated cells. PKR is activated by type I and type III IFNs and exerts its antiviral effects via phosphorylating the eukaryotic translation initiation factor (eIF2). Phosphorylation of EIF2 causes the sequestration of eIF2b, resulting in the blockage of the conversion of GDP to GTP, which subsequently suppresses the translation [135].

Certain ISGs, including tetherin, can affect viruses at the post-translation stage. It possesses a distinct structure, with transmembrane anchors at both ends of the protein lodged in the cellular membrane. After being encoded BST2 gene, tetherin blocks viral proteins from exiting infected cells [136]. In this section, we will review the antiviral effects of the JAK/STAT signaling pathway on the pathogenesis of different viral infections.

#### Human immunodeficiency virus (HIV)

The immune system adapts complex innate and adaptive defense mechanisms against HIV. For instance, IFITM-3 is regarded as a substantial viral restriction factor in the course of HIV infection. Genetic analysis has shown that a variation in the IFITM-3 gene is substantially more common in children infected via mother-to-children transmission than in uninfected children [137]. HIV-1 infection is known to be markedly mitigated by MX2/MxB. Results of a new study displayed that the existence of aspartic acid at Ser28, Thr151, or Thr343 resulted in increased antiviral activity: these are known as hypermorphic mutants. In certain circumstances, these hypermorphic alterations gained the ability to suppress HIV-1 capsid variants that are thought to be resistant to wild-type MX2 (e.g., S28D/T151D or T151D/T343A) [131]. Tetherin can block retroviral replication in vivo through an IFN-dependent mechanism. Arias et al. [138] highlighted the role of viral protein U (Vpu) protein, which is known to degrade tetherin. They reported that Vpu mutations increased the susceptibility of the HIV-1 virus to antibody-dependent cell-mediated cytotoxicity, implying that tetherin serves as a link between innate and adaptive immune defense against the virus. Another assay of JAK/STAT signaling in cells of HIV-1-infected individuals confirmed a particular defect in GM-CSF-stimulated STAT5 phosphorylation in the monocytes of the donors. Conversely, evaluation of GM-CSF-stimulated MAPK pathways showed elevated levels of lipopolysaccharide-stimulated extracellular signal-regulated kinase phosphorylation in monocytes [139].

#### Severe acute respiratory syndrome coronavirus 2 (SARS-CoV-2)

During the COVID-19 pandemic, many researchers tried to explain the role of the JAK/STAT pathway in the course of infection as well as its potential diagnostic and therapeutic applications. In SARS-CoV-2 infection, inflammation occurs via the JAK/STAT cascade causing the activation of diverse cells, including macrophages, lymphocytes, dendritic cells, and natural killer cells. This process eventually results in a cytokine storm. Immune responses via B cell and T cell differentiation are also mediated by this signal [5]. Rincon-Arevalo and colleagues confirmed that in COVID-19 patients with mild and severe involvement, STAT1 and IRF9 are upregulated, which is parallel to the IFN signature examined by the expression of CD169 on peripheral monocytes. Meanwhile, in cases of severe infection, the expression of CD169 and STAT1 in plasmablasts and CD14 + monocytes were negligible compared to that of milder cases [140]. Quantitative assessment of the activity of the JAK/STAT cascade has been used to estimate innate and adaptive immune responses to COVID-19 infection. JAK-STAT1/2 help to distinguish between virus and bacteria, determining the extent of the adaptive cellular immune response. Also, JAK-STAT3 can serve as a predictor of cytokine storm syndrome [141]. In the case of therapeutic strategies, the potential effectiveness of JAK1 and JAK2 inhibitor ruxolitinib was suggested in terms of preventing cytokine storm [142].

#### Influenza

STAT3 is necessary for Influenza replication suppression. Mahony and colleagues [143] showed that STAT3 shRNA knockdown increased influenza replication while inhibiting the induction of numerous well-studied antiviral ISGs: PKR, OAS2, MxB, and ISG15. The findings of a recent study suggested that infection with the influenza A virus significantly augmented the expression of the long noncoding RNA (lncRNA) IFITM4P. The lncRNA IFITM4P appeared to be an effective modulator of innate antiviral immunity. In vitro, ectopic expression of the lncRNA IFITM4P dramatically inhibited IAV replication, while IFITM4P depletion increased virus generation. Also, IFN signaling boosted the expression of the lncRNA IFITM4P during influenza virus infection, and the altered expression of this lncRNA had a substantial influence on the mRNA levels of multiple IFITM family members, including IFITM1, IFITM2, and IFITM3 [132].

#### Other viruses

The antiviral properties of the JAK/STAT pathway have also been explained in other viral infections. In African swine fever disease, which arises from involvement by the African swine fever virus, STAT1 and STAT2 signaling enhance IFN-β activation to exert antiviral effects against the pathogen [144]. In an animal study, a novel JAK gene (LvJAK) was reported to be involved in antiviral immune defense against the white spot syndrome virus in *Litopenaeus vannamei* [145]. In a Drosophila model, it was observed that infection with the invertebrate iridescent virus 6 induces JAK/STAT signaling via the expression of a gene family of IL-6-related cytokines [146].

Despite the identified effects of the JAK/STAT cascade on antiviral immunity, it is noteworthy that several viruses have established strategies to confront this system. At the same time, researchers have tried to discover these antagonizing mechanisms and intervene in them to produce novel antiviral medications and vaccines. To give an instance, Devaux and co-authors [147] introduced a recombinant measles virus that lacked the ability to antagonize STAT1 activities. The virulence of this recombinant virus was examined in rhesus monkeys, and none of them developed the symptoms typically seen with the wild-type virus. It was revealed that cross-talks between measles and STAT1 are essential for the virus to retain its virulence. Additionally, the administration of the recombinant virus led to an immune response typical of the wild-type virus, indicating its potential for vaccination (Table 3).

**Table 3:** Overview of the strategies implemented by viruses to evade the JAK/STAT cascade

Mechanism	Virus applying the mechanism	JAK/STAT component affected	Evasion mediator	Outcome
Suppression of IFN signaling	West Nile virus	STAT1	NS5	Antagonizing type I IFN-mediated JAK-STAT signaling [32]
	Kaposi's sarcoma-associated herpesvirus	JAK1, TyK2, and STAT2	RIF	Blockage of IFN signaling by producing inhibitory complexes containing IFNAR subunits [33]
	Herpes Simplex Virus 1	JAK1	UL36USP	Suppression of IFN-β-induced activation of ISRE promoter and transcription of ISGs [34]
Promotion of proteasomal degradation through ubiquitination	Hepatitis C virus	STAT3	Direct ubiquitination of STAT3	Inhibition of IFN-α responses [35]
	HIV	STAT1 and STAT3	Vif	Blockage of IFN-α functions [36]
	Porcine reproductive and respiratory syndrome virus	STAT2	Nsp11	Inhibition of IFN antiviral activity [37]
Inhibition of STAT phosphorylation	Japanese encephalitis virus	STAT1 and Tyk2	Protein tyrosine phosphatases	Suppression of the induction of IFN-stimulated genes and the antiviral function of IFN-α [38]
	Human papilloma virus	STAT1, STAT2, and Tyk2	Direct physical interaction with the receptor-associated kinases	Blocking the antiviral function of IFNs [39]
	Sendai virus	Tyk2, STAT1, STAT2, and STAT3	C proteins	Inhibition of the antiviral function of IFN- α [40]
	Varicella zoster virus	STAT2	ORF63	Resistance to IFN treatment [41]
Blockage of the nuclear transport of ISGF3	PRRSV	STAT1 and STAT2	Nsp1β	Suppression of the IFN-mediated antiviral responses [42]
	Porcine epidemic diarrhea virus nsp7	STAT1 and STAT2	Interaction with the DNA binding domain of STAT1/STAT2	Suppression of type I IFN signaling pathway [43]
	Herpes simplex virus type 2	STAT1 and STAT2	ICP22	Interference with IFN type 1 signaling [44]
Upregulation of SOCS	Influenza A virus	JAK1	Upregulating mRNA level of SOCS1	Antagonizing type I and type II IFN responses [45]
	Enterovirus 71	STAT3	Upregulating mRNA level of SOCS1 and SOCS3	Downregulation of the JAK/STAT signaling pathway in an interferon-independent way [46]

*IFN* Interferon, *HIV* Human immunodeficiency virus, *PRRSV* Porcine reproductive and respiratory syndrome virus, *SOCS* Suppressor of cytokine signaling, *ISRE* Interferon-sensitive response element, *ISGs* Interferon-stimulated genes

### The effect of the two edges of the JAK/STAT pathway (antivirus or with virus)

**Fig. 2:**
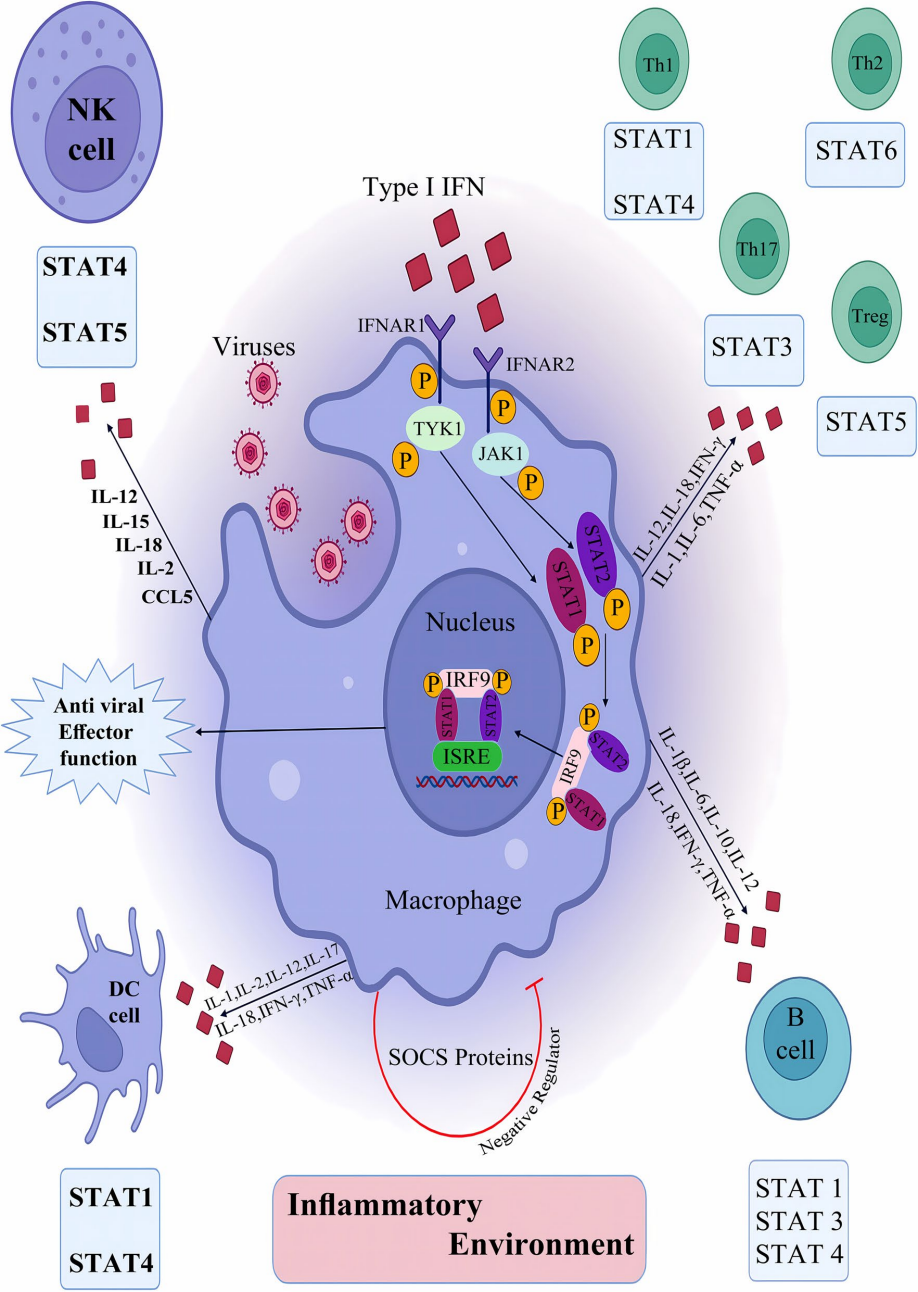
Double-edged role of JAK/STAT signaling cascade in viral infections

Despite the role of the JAK/STAT in controlling immune responses against viral pathogens, recent evidence points to the positive effects of this cascade on the replication and pathogenesis of viral infections both in animals and humans. Pro- and antiviral activities of different components of the JAK/STAT signaling cascade have been confirmed; however, the exact mechanisms of these activities are yet to be investigated [13]. The role of the JAK/STAT pathway in suppressing or promoting viral infections has been widely examined and has revealed controversial results (Fig. 2). Mahony and colleagues have shown that STAT3 is essential for suppressing Influenza and Vaccinia replication in the IFN-α pathway in human hepatocytes [143]. In highly pathogenic avian influenza H5N1 virus infection of chickens, the raised pro-inflammatory response is mediated by the suppression of STAT-3 and is implicated in the pathogenicity of infection [148]. While IFN regulatory factor 3 and STAT1 show powerful antiviral function against varicella-zoster virus (VZV), this virus has been reported to induce STAT3 phosphorylation in cells involved in vitro and in vivo in a mouse model, leading to enhancing the virus replication as well as skin pathogenesis [149]. The mumps virus V protein has been demonstrated to target STAT1 and STAT3 for degradation. Modification of this targeting by inducing a single-point mutation showed that STAT3 was a mediator of anti-mumps responses [150]. In the case of hepatitis viruses, STAT3 is regarded as a substantial factor for hepatitis B virus (HBV) gene expression. It is also involved in liver cancers, playing a substantial role in tumor formation [151]. Moreover, the hepatitis C virus (HCV) triggers activation of STAT-3 via oxidative stress and cellular kinases, namely p38 MAPK, JAK-2, Src, and JNK. The activated STAT-3 subsequently contributes to HCV RNA replication [152]. Also, lncRNAs, including lncIGF2AS and lnc7SK, are upregulated by STAT3, participating in HCV replication [153]. In the case of hepatitis *Delta* virus (HDV), MOV10 has been demonstrated to interact with HDAg. HDV replication is hindered when MOV10 expression is suppressed. Moreover, the small isoform of adenosine deaminase acting on RNA (ADAR 1) can modify the HDV RNA antigenome. This process can be suppressed by HDAg-S, limiting the synthesis of HDAg-L and controlling the phases of the HDV life cycle. Therefore, ADAR 1 is a critical host protein required for HDV infection development [154]. A study on hepatocellular carcinoma cells confirmed that STAT3 signaling is involved in hepatitis E virus replication [155]. Regarding SARS-CoV-2 infection, STAT-3 is involved in promoting inflammatory responses during the course of the disease. On the other hand, it can inhibit antivirus responses and induce uncontrolled antivirus adaptive immune responses by affecting Treg-, Th1-, Th17-, and B cell-mediated processes [156].

Overall, components of the JAK/STAT pathway are a determining factor in the complex interactions between viral pathogens and their hosts, exerting either pro- or antiviral effects depending on the type of virus and host cell. Some researchers have used this concept to introduce potential antiviral interventions by modifying the genes and molecules involved. Relevant clinical data on the use of JAK inhibitors in COVID-19 patients, including baricitinib, ruxolitinib, tofacitinib, and nezulcitinib, suggest the advantages of this drug class in regards to risk reduction for major outcomes when combined with the routine regiment of COVID-19 patients [157]. Given the role of the JAK/STAT in the pathogenesis of certain oncogenic viruses, these strategies can be beneficial in the field of cancer therapy. Blockage of STAT3 by shRNAs has shown excellent results in suppressing the progression of HBV-related hepatocellular carcinoma (HCC) [80]. Similarly, blockage of STAT3 activation has resulted in a drop in the HEV ORF2 protein concentrations in HCC cells [155]. In T‑cell lymphoma, the findings have indicated the involvement of IL‑6/JAK/STAT3 signaling. Meanwhile, transglutaminase 2 siRNAs are proposed as a potential therapeutic choice for blocking the proliferation of lymphoma cells by suppressing this signaling pathway [158].

## Conclusion

As discussed in this article, JAK/STAT signaling pathway and its related cytokines are substantial for host defense against viral pathogens. However, in some viral diseases, they might exert pro-viral effects, leading to the progression of the disease. Novel research has resulted in fundamental updates in our knowledge of the cross-talk between this cascade and immune and antiviral responses. Despite these advancements, there are still substantial questions on determining factors in the switch between pro- and antiviral functions of the JAK/STAT and the exact mechanism through which viruses exploit this cascade for their gene replication and pathogenesis. Also, certain challenges remain about the potential of JAK inhibitors as antiviral agents, including the types of viral diseases that could be treated with the JAK/STAT inhibitors. Furthermore, the administration of JAK inhibitors has been associated with some complications, such as immune suppression. Therefore, further preclinical and clinical research is needed to comprehensively examine the specific antiviral or pro-viral activities of JAK/STAT components in each viral infection. Another existing challenge is to determine the efficient dose of JAK/STAT inhibitors for different acute and chronic viral diseases.

## Data Availability

Not applicable.
